# Zinc-deficient diet aggravates ventilation-induced lung injury in rats

**DOI:** 10.1016/S1674-8301(12)60008-6

**Published:** 2012-01

**Authors:** Xiaoyu Chen, Jieyu Bian, Yingbin Ge

**Affiliations:** a Department of Pediatrics, the First Affiliated Hospital to Nanjing Medical University, Nanjing, Jiangsu 210029, China;; b Department of Physiology, Nanjing Medical University, Nanjing, Jiangsu 210029, China.

**Keywords:** ventilation, lung injury, zinc deficient, nuclear factor-κB (NF-κB), vascular endothelial growth factor (VEGF), rat

## Abstract

We investigated the effects of zinc deficiency on acute lung injury (ALI) induced by mechanical ventilation. Male Sprague-Dawley rats were fed with a zinc-deficient or zinc-proficient diet for 4 weeks, and then received mechanical ventilation at normal frequency and pressure for 30 min. Total protein, cell count, the number of polymorphonuclear neutrophil (PMN) in the bronchoalveolar lavage (BAL), and vascular endothelial growth factor (VEGF) expression in the lung were determined. Activation of nuclear factor-κB (NF-κB) was detected by examining the phosphorylation of NF-κB (pNF-κB p65) and the expression of inhibitor of NF-κB (pI-kBα). Compared to the controls, total cell count and the number of PMNs were significantly increased to 160% and 140%, respectively, in zinc-deficient rats treated with ventilation. Activation of NF-κB was significantly increased and VEGF was also increased to three-folds. Zinc deficiency aggravated the inflammatory response in rats and was associated with the overexpression of VEGF in response to mechanical ventilation. Zinc supplementation may be beneficial to zinc-deficient patients during mechanical ventilation.

## INTRODUCTION

Mechanical ventilation is a life-support modality for respiratory failure patients in infant care unit and intensive care unit[1],[2]. In the process of mechanical ventilation, over-distention of diseased alveoli by positive pressure promotes inflammation and further disrupts the alveolocapillary membrane, resulting in capillary stress failure, plasma membrane microrupture and lung cell death[3],[4]. Acute lung injury (ALI) is a form of pulmonary edema due to increased microvascular permeability, and could result from mechanical ventilation. It is a major cause of respiratory morbidity and mortality.

Maintaining end-expiratory lung volume at limited tidal volume with positive end-expiratory pressure (PEEP) can prevent or reduce ventilator-induced lung injury[5]-[7]. Therefore, the basic therapy of ALI combines a pressure-limited lung protective mechanical ventilation with low tidal volumes (6-8 mL/kg ideal body weight). Adequate PEEP has been shown to reduce the release of systemic inflammatory mediators, increase ventilator-free days and decrease mortality. However, complications of ventilator injury may still occur with substantial frequency in this therapeutic modality during mechanical ventilation[8] and the factors involved still remain unclear.

Zinc is an essential dietary micronutrient with beneficial functions that facilitate cytoprotection, improve wound healing, and promote tissue repair[9]. Many proteins and biological enzymes contain zinc, including zinc finger proteins[10], alkaline phosphatase, alcohol dehydrogenase, aspartate transcarbamylase, copper/zinc superoxide dismutase, and angiotensin I-converting enzyme[11]. Zinc deficiency induces a significant pathological state, which includes intestinal[12], dermatological[13], neurological[14], and immunological[15] problems. For example, zinc deficiency increases organ damage and mortality in a murine model of polymicrobial sepsis[16].

We hypothesized that zinc deficiency is associated with significantly increased lung injury during mechanical ventilation. To test this hypothesis, we measured the alveolar capillary permeability in the lungs of mechanically ventilated zinc-deficient rats without previous lung injury. Possible mechanisms responsible for the augmented lung injury were also investigated.

## MATERIALS AND METHODS

### Animals

Male Sprague-Dawley rats (100 g) were obtained from Nanjing Medical University Animal Centre, and acclimated for 1 week. Rats were housed individually at (22±2)°C under a 12-h light–dark cycle. The experimental protocols were compliant with “Guide for the Care and Use of Laboratory Animals” published by the Chinese National Institutes of Health, and approved by the Animal Ethics Committee of Nanjing Medical University.

### Zinc-deficient rat model

Rats were placed under a zinc-deficient diet (0% zinc content) or control diet (0.01% zinc content) for 4 weeks[17]. The two diets were identical in protein, carbohydrates, fat, vitamins, and minerals (except zinc). To maintain an isocaloric intake in the two groups, the same amount of diet was given every day.

### Measurement of serum Zn

Serum Zn was measured using atomic absorption spectrophotometry (Z-6100, Hitachi, Tokyo, Japan), as previously reported[18]. Whole blood was removed from the vena cava under diethyl-ether anesthesia, and centrifuged at 1,000 *g*. The centrifuge tubes had been washed with acid solution to remove trace metal contamination. The obtained serum was transferred into a washed tube, and diluted using deionized water. Diluted sample was aspirated into an atomic absorption spectrophotometer to determine absorption at 213.9 nm.

### Measurement of lung metallothionein content

Lung tissues were homogenized in 10 mmol/L Tris-HCl buffer, pH 7.4, at 4°C. After centrifugation of the homogenate at 10,000 *g* for 15 min, 200 µL supernatant was transferred to microtubes for metallothionein (MT) analysis, and 100 µL was transferred to separate microtubes for protein analysis. Samples were then prepared for MT determination using a cadmium–hemoglobin affinity assay, as described previously[19].

### Mechanical ventilation

Mechanical ventilation was perform as described previously[20]. Intubation was carried out under anesthesia with 2% isoflurane in 65% nitrogen dioxide/35% oxygen. A sterile polyethylene catheter was placed in the carotid artery for drawing arterial blood samples and monitoring blood pressure. A sterile metal tube was placed in the trachea. After these surgical procedures, gaseous anesthesia was discontinued and anesthesia was maintained with 60 mg/kg pentobarbital sodium intraperitoneally. (Sigma, St. Louis, MO, USA). Muscle relaxation was induced by pancuronium bromide 2 mg/kg, i.m. (Sigma). Ventilation was carried out with an ASV0691-001 ventilator (Havard, USA) in a pressure-constant time cycled mode and an inspiratory oxygen fraction of 1.0.

Zinc-deficient and proficient diet rats (4 rats in each group) were mechanically ventilated at a frequency of 30 breaths/min with normal pressure (13/3; PIP/PEEP in cmH_2_O) for 30 min. Ventilation using these parameters does not cause lung injury in normal rats, as previously reported[20]. Groups of zinc-deficient and normal diet rats not subjected to mechanical ventilation were also included as controls. At the end of the experiment, heparinized arterial blood was collected, and animals were sacrificed with an overdose of pentobarbital.

### Bronchoalveolar lavage

Bronchoalveolar lavage (BAL) was performed with 5×10 mL of prewarmed (37°C) sterile 0.9% saline via the tracheal tube. The fluid was aspirated with a disposable pyrogen-free syringe and immediately centrifuged (10 min, 4°C, 1,000 *g*). Total protein in bronchoalveolar lavage fluid (BALF) was measured by the bicinchoninic acid method (BCA assay; Pierce, Rockford, IL, USA). The cell pellet in BALF was washed with phosphate buffered saline (PBS) twice, and the cells were counted with a hematocytometer. Cytocentrifuged preparations were stained with a modified Wright-Giemsa method, and differential cell count, based on morphologic criteria, was carried out on 200 consecutive cells. The number of polymorphonuclear neutrophils (PMNs) in BALF was calculated by multiplying the ratio of PMNs with total cell count.

### Western blotting analysis

Lung tissues were homogenized in a lysis buffer containing 50 mM Tris–HCl (pH 7.5), 150 mmol/L NaCl, 5 mmol/L EDTA, 10 mmol/L NaF, 1 mmol/L sodium orthovanadate, 1% Triton X-100, 0.5% sodium deoxycholate, 1 mmol/L phenylmethylsulfonyl fluoride and a protease inhibitor cocktail. The lysate was centrifuged at 12,000 g for 25 min at 4°C. The total protein in each sample was analyzed by the BCA method (Pierce). An equal amount of protein samples, 60 µg, from each animal was boiled in 3×loading buffer (10 mmol/L Tris-HCl, pH 6.8 including 3% SDS, 5% β-mercaptoethanol, 20% glycerol and 0.6% bromophenol blue) for 3 min and separated by 12.5% SDS-polyacrylamide gel electrophoresis (SDS-PAGE) and transferred to nitrocellulose membranes. After the transfer, membranes were blocked with 5% fat-free milk in Tris-buffered saline plus 0.05% Tween 20 overnight at 4°C. The membranes were then incubated with a rabbit anti-VEGF (Santa Cruz Biotechnology, Santa Cruz, CA, USA), rabbit anti-phosphorylated IκB-α/β, p-IκB, p65, p-p65 (Santa Cruz Biotechnology) or rabbit anti-β-actin antibodies (Sigma) for 2 h at room temperature. After washing in TBST for three times, the membranes were incubated with a peroxidase linked anti-rabbit-IgG conjugate for 1 h at room temperature. Finally, they were washed again in TBST and incubated in enhanced chemiluminescence reagents (Pierce).

The intensity of the bands were quantified for 2 min, and exposed to X-Omat BT film. Signal intensity was quantified using a Bio-Rad image analysis system. The results were normalized using β-actin as internal control. For background, the primary antibody was omitted.

### Immunohistochemistry

The tissues from the right lobe of the lung were fixed in 10% formaldehyde for 24 h at room temperature. Tissue specimens were cut into 6-µm-thick slices. The slices were deparaffinized in xylene, dehydrated in gradient ethanol, and then washed with TBS. For heat-induced epitope retrieval, the sections were dipped in 10 mmol/L citrate buffer (pH 6.0) and heated with a microwave oven for 15 min. After pretreatment in 1% H_2_O_2_ in PBS for 30 min and blocking with 5% BSA at room temperature, sections were incubated with anti-VEGF (Santa Cruz Biotechnology) overnight at 4°C. After the primary antibody reaction, an immunohistochemical staining kit (DAKO, CA, USA) was used. The slides were washed and stained with 3,3′-diaminobenzidine and then counterstained with hematoxylin. Photographs of the tissue specimens were taken at ×200 magnification.

### Statistical analysis

All experiments were done in triplicate. Analysis of the experimental data was carried out using the PDQuest 7.0 software (Bio-Rad Laboratories, Hercules, CA, USA). Data were analyzed with one-way analysis of variance, followed by Bonferroni correction. Data were presented as mean±SD. *P* < 0.05 was considered statistically significant.

## RESULTS

### Comparison between zinc-deficient and proficient rats

Zinc-deficient diet for four w resulted in the development of characteristic alopecia around the thigh. Serum zinc concentration was (27.6±5.1) µg/dL in the zinc-deficient animals and (111.2±8.9) µg/dL in the control group (*P* < 0.05). A concomitant decrease in lung tissue content of metallothionein occurred in the zinc-deficient animals [(8.5±0.12) µg/dL compared to that of controls (11.2±0.9) µg/dL, *P* < 0.05]. Zinc-deficiency also resulted in slower gain of body weight [(150.3±5.8) g in zinc-deficient animals *vs* (187.1±6.9) g in controls after 4 w; starting weight = (120.3±4.8) g].

### Mechanical ventilation induces lung injury *in vivo*

We ventilated Sprague-Dawley rats with 13/3 cm H_2_O (PIP/PEEP) with a tidal volume of 18 mL/kg. Ventilation with 13/3 cmH_2_O for 30 min was well tolerated as indicated by normal blood oxygenation. Serum transaminase and creatinine levels were not different in ventilated and non-ventilated animals (data not shown). Mechanical ventilation resulted in an increase of total cell count in the BALF, and significant neutrophil recruitment to the alveoli in zinc-deficient rats. We also observed increased protein content in the BALF following 30-min mechanical ventilation in zinc-deficient rats (***Table 1***).

### Expression of VEGF

**Fig. 1:**
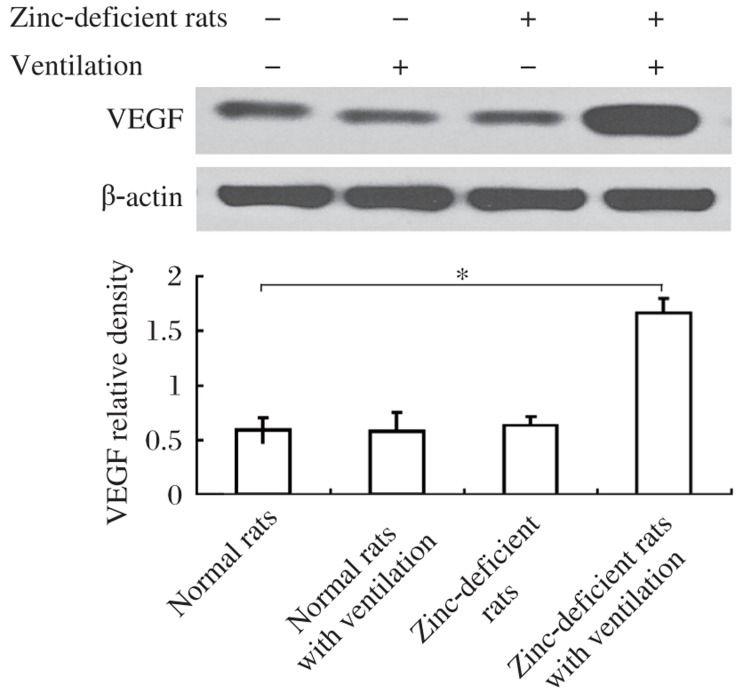
Effect of ventilation on the expression of VEGF in rat lung tissue by Western blotting. The ventilation with 13/3 cmH_2_O resulted in a three-fold production of VEGF after 30 min in zinc-deficient rats than those in normal animals or zinc-deficient rats without ventilation. A: representative Western blots from rats treated with or without ventilation for 30 min. B: results of densitometric analysis (*n* = rats per group), normalized against β-actin. Data are expressed as fold increase over the basal condition, *P < 0.05.

**Fig. 2:**
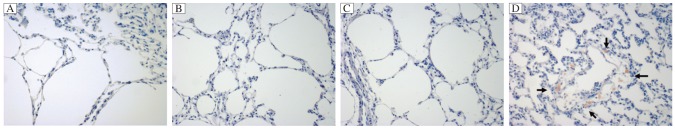
Effect of ventilation on the expression of VEGF in rat lung tissue by immunohistochemistry (Original×200). No VEGF positive cell was found in normal animals (A, B) or zinc-deficient rats without ventilation (C). The VEGF positive cells around the alveolar blood vessel are indicated by arrows in zinc-deficient rats ventilated 30 min with 13/3 cmH_2_O (D).

Ventilation with 13/3 cmH_2_O for 30 min increased VEGF content in the lung by three fold in zinc-deficient rats (***Fig. 1***). This result was confirmed by im-munohistochemical staining: VEGF was expressed mainly in alveolar epithelial cells in the lungs of zinc-deficient rats (***Fig. 2***).

### NF-κB activation

**Fig. 3:**
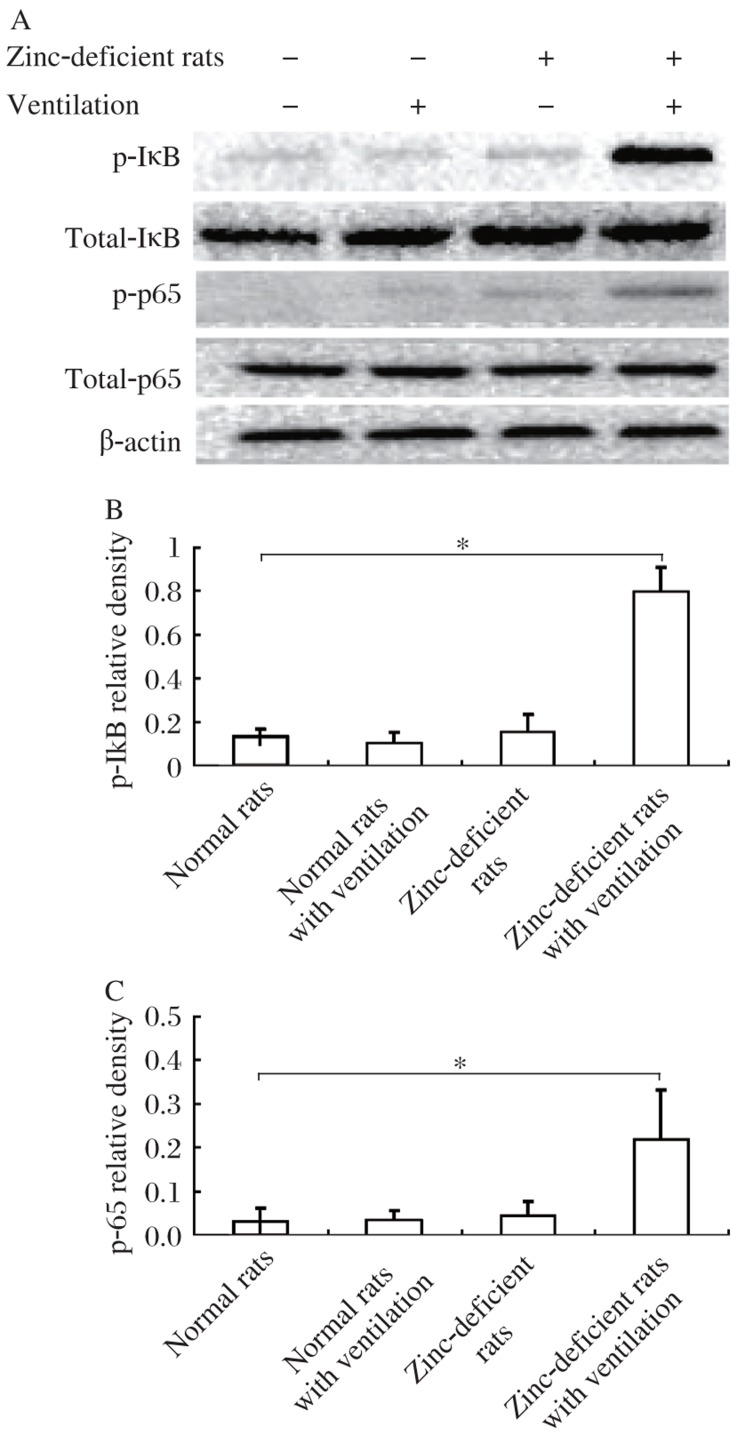
Effect of ventilation on the activation of NF-κB in rat lung tissue by the expression of phosphorylation of I-κBα and NF-κB p65 using Western blotting. I-κBα and p65 were detected using antibodies specific for phosphorylated or unphosphorylated forms of I-κBα and p65. Ventilation with 13/3 cmH_2_O rapidly increased phosphorylation of Iκ-Bα and NF-κB in zinc-deficient rats with ventilation. Densitometric analysis was carried out of five different gels of I-κBα (B) or p65 (C), normalized against β-actin. Data are expressed as fold increase over the basal condition, *P < 0.05.

The activation of NF-κB ERK was measured by the expression of phosphorylation of NF-κB (pNF-κB p65) and the I-κBα by Western blotting in lung tissue homogenate. Ventilation with 13/3 cmH_2_O rapidly increased phosphorylation of the I-κBα in zinc-deficient rats. Phosphorylation is known to lead to degradation of Iκ-Bα, which in turn facilitates nuclear translocation of NF-κB. The increase of NF-κB phosphorylation demonstrated that NF-κB was activated by translocation from the cytoplasm to the nucleus (***Fig. 3***).

## DISCUSSION

Zinc deficiency is known to induce alopecia. Cui *et al*.[21] reported that rats fed with a zinc-deficient diet for one week developed dermatitis of the extremities, balanitis, stomatitis, and alopecia. Characteristic alopecia and significantly reduced Zn concentration in serum and lung tissue levels were observed under our experimental conditions, suggesting that our rat model corresponds to mild zinc deficiency.

Mechanical ventilation could cause inflammatory infiltration, pulmonary edema, hyaline membranes, and surfactant dysfunction in the lungs[22]. Previous studies have shown that ventilation itself may not cause extensive lung injury under normal conditions, but could augment lung inflammation in preinjured lungs[23],[24]. Similar to the findings from a previous study[20], our results showed that mechanical ventilation for 30 min with 13/3 cmH_2_O (PIP/PEEP) does not cause lung injury under normal condition. However, mechanical ventilation in zinc-deficient rats resulted in significant damage to the lung, increased pulmonary permeability, and enhanced neutrophil recruitment (***Table 1***).

**Table 1:** Effect of mechanical ventilation on lung injury in rats

Group	Total protein (mg/mL)	Total cells (10^3^)	Percentage of neutrophil (%)
Normal rats	0.69 ± 0.03	23.00 ± 1.50	51.00 ± 2.30
Normal rats with ventilation	0.71 ± 0.02	21.00 ± 2.10	49.00 ± 1.80
Zinc-deficient rats	0.67 ± 0.01	26.00 ± 1.10	53.00 ± 2.40
Zinc-deficient rats with ventilation	1.19 ± 0.06*	37.00 ± 2.70*	71.00 ± 4.30*

*Compared to normal rats, *P* < 0.05.

In addition to pulmonary edema, mechanical ventilation can also cause biotrauma[25] through the initiation of an inflammatory response, such as the release of numerous cytokines and chemokines[26],[27]. Activation of inflammatory pathways following these intracellular processes, most notably the activation and translocation of NF-κB, is thought to be a key event in stretch-induced lung injury *in vivo*[28]. NF-κB is bound to and inhibited by the NF-κB-inhibitory protein family composed of I-κBα, I-κBβ, and I-κBϵ. Upon stimulation, the I-κB kinase complex is activated, resulting in the phosphorylation of I-κBs by the I-B kinase (IKK) complex[29]-[31] at two conserved *N*-terminal serine residues and NF-κB activation on nuclear accumulation and transactivation potential. The IKK complex includes IKKβ, IKKα and NF-κB essential modulator (NEMO) or IKKγ. IKKγ itself does not have kinase activity, but is absolutely required for such a canonical pathway of NF-κB activation, likely by acting as a scaffold protein in the IKK complex, and is essential for I-κB kinase activation[32],[33]. IKKγ contains several putative functional domains, including a leucine zipper motif and a putative zinc finger domain at the C terminus. Mutations in the coil-zipper (CoZi) domain of IKK can cause signaling defects or constitutive NF-κB activity[32]. In the present study, we found increased NF-κB activation in the lung tissues in zinc-deficient rats exposed to mechanical ventilation. It is likely that zinc deficiency promotes degradation of I-κB kinase and NF-κB activation through the zinc-finger domain of IKKγ. A previous report observed that NF-κB activation is significantly increased in the lung as a consequence of zinc deficiency and thereby amplifies innate activation during the early stages of sepsis[34]. Our findings represent an extension of previous observations.

Vascular permeability factor, also known as vascular endothelial growth factor (VEGF) and its receptor are very important in lung angiogenesis, development, and function maintenance, and could be upregulated by NF-κB activation[35]. VEGF is involved in vascular basement membrane destruction and angiogenesis, and is a potent mediator of capillary leak if it gains access to its receptors on the capillary endothelium. Overexpression of VEGF has been demonstrated to induce formation of fenestrations and thin endothelial cell cytoplasm may allow leak of solutes, resulting in pulmonary edema[36],[37]. In zinc-deficient rats, there was a marked increase of VEGF following mechanical ventilation. Immunohistochemistry results confirmed the abundant presence of VEGF peptide in the lung parenchyma in the zinc-deficient animals under mechanical ventilation. Both Western blotting and immunohistochemistry results indicated that ventilator-induced lung injury in zinc-deficient rats involves VEGF and NF-κB activation.

Recruitment of inflammatory cells to the lung is also linked to the activation of NF-κB[38]. The role of activation of NF-κB in cytokine release linked to the recruitment of inflammatory cells to lung injury in zinc-deficient rats was not investigated. This is a limitation of the present study. We plan to answer these questions by using samples from animal models or human patients with ventilator -induced lung injury with or without zinc-deficiency in the near future.

Taken together, we demonstrated for the first time that zinc deficiency contributes to the pathogenesis of lung injury in response to mechanical ventilation by enhancing the activation of NF-κB pathways and overexpression of VEGF. It is likely that a clinically significant number of patients in intensive care unit are zinc deficient[39]. A preliminary study indicated that zinc supplementation administered before sepsis could promote survival and normalization of lung pathology[34]. Short-term zinc supplementation may ameliorate inflammatory responses in zinc-deficient patients receiving mechanical ventilation.
